# Association of liver biomarkers and cytokeratin-18 in Nonalcoholic fatty liver disease patients

**DOI:** 10.12669/pjms.36.3.1674

**Published:** 2020

**Authors:** Benash Altaf, Anam Rehman, Shireen Jawed, Abdul Raouf

**Affiliations:** 1 Dr. Benash Altaf, MBBS, M.Phil. Assistant Professor, Department of Physiology, Aziz Fatimah Medical and Dental College, Faisalabad, Pakistan; 2 Dr. Anam Rehman, MBBS, M.Phil. Senior Demonstrator, Department of Physiology, Aziz Fatimah Medical and Dental College, Faisalabad, Pakistan; 3 Dr. Shireen Jawed, MBBS, M.Phil. Associate Professor, Department of Physiology, Aziz Fatimah Medical and Dental College, Faisalabad, Pakistan; 4 Dr. Abdul Raouf, Assistant Professor, Department of Radiology, Allied Hospital, Faisalabad Medical University, Faisalabad, Pakistan

**Keywords:** Cytokeration18, Liver biomarkers, Non-Alcoholic fatty liver disease

## Abstract

**Objective:**

To investigate the association of gold standard liver biomarkers with serum cytokeratin 18 (CK18), serum Alanine aminotransferase (ALT) and serum aspartate (AST).

**Methods:**

This was cross sectional study. It was conducted at Mayo Hospital from January 2016 to December 2017. It comprised of 148 non-alcoholic fatty liver disease subjects of age 40-60 years. After written informed consent, study anthropometric measurements (age, height, waist circumference and hip circumference) were taken and serum AST, ALT and CK-18 were estimated by sandwiched ELISA technique. Data was analyzed using SPSS 21.0. Descriptive were presented as mean and standard deviation. Association between CK18, serum AST and ALT were analyzed by regression analysis and are presented as beta coefficient. P-value ≤ 0.05 was taken as significant.

**Results:**

Study comprised of 148 subjects with mean age 44.81±6.2. Of total population 29.1% were male and 70.9% were female. Significant positive association of CK18 was found with serum ALT (P-value 0.005*). However, no association was found between AST and serum CK18. (P-value 0.29).

**Conclusion:**

Significant positive association was found between Serum CK18 and serum ALT.

## INTRODUCTION

Non-alcoholic fatty liver disease (NAFLD) is becoming prevalent due to progressive increase in prevalence of obesity and diabetes. It is involving more than one fourth of the adults globally with 25% prevalence rate.^1^ NAFLD is one of the utmost common reasons of chronic liver diseases leading to complications like simple steatosis, non-alcoholic steatohepatitis (NASH), fibrosis, and eventually hepatocellular carcinoma (HCC) which is the foremost indication for liver transplantation.^2^ Hence, timely diagnosis and disease progression is important in order to avoid life threatening complications. For diagnosis, ultrasonography, computed tomography (CT) are helpful but liver biopsy is considered as the gold standard.

On histology, NAFLD is depicted by the presence of hepatic steatosis which is excess lipid accumulation without any significant inflammation. Whereas NASH is well defined histologically by the presence of hepatic steatosis along with the inflammation of lobules and the hepatocellular degeneration with or without the pericellular fibrosis.^3^

NASH comprises of a spectrum of pathological changes in liver including apoptosis, fibrosis leading to cirrhosis or the hepatocellular carcinoma that involves 20% of the population. Inspite of NAFLD being epidemic in chronic liver disease, the exact diagnostic options are still needed, so adding up the economic burden on the system of healthcare.^2^

Currently, liver biopsy is the gold standard for NAFLD diagnosis and to assess its progression. However, this procedure has some significant restrictions. First of all, it is invasive requiring hospitalization. Secondly, it might result in crucial life threatening complications like hemorrhage etc. Furthermore, ethically it is not possible to perform biopsy repeatedly in NAFLD subjects to see the disease progression. The relative complications and high expenses of gold standard test utilized for the diagnosis of NAFLD has insisted the field to pursuit for more diagnostic methods.^4^ Evidence based researches have suggested that serum biomarkers including serum AST, ALT and CK18 have a remarkable role in early detection of the liver diseases.^5^ Elevation of serum transaminase levels in NAFLD patients has been well documented by previous studies. On the other hand, serum ALT is generally higher than AST in diseased liver conditions. However, some recent studies were not in agreement with these findings. Many patients with advance fibrosis and NASH can also present with normal liver enzymes.^6^

CK18 is present in huge amount in liver. It is an intermediate filament protein representing 5% of the hepatic proteins. In the apoptosis of cell, CK18 is cleaved and the fragments of CK18 are identified by M30 antibody specific for liberated C-terminus. Aim of the study was to find the association of liver biomarkers serum AST and ALT with CK18 which can be used as diagnostic biomarker as well as marker for disease progression in future.

## METHODS

This was a cross sectional study, conducted at outpatient Department of Radiology of Mayo Hospital, King Edward Medical University, Lahore, after taking ethical approval (134/RC/KEMU) from January 2016 to December 2017. Total 148 NAFLD subjects were recruited by non-probability convenient sampling technique. NAFLD subjects with fatty liver were diagnosed on ultrasonography with high echogenic shadows. Known diabetic subjects, subjects suffering from any type of hepatitis, taking homeopathic medications or glucocorticoids and taking alcohol were excluded from the study. Non-diabetic subjects of both gender of age ranging 40-60 years with fatty liver were included in this study. Pre-screening was done for fasting blood sugar levels with the help of glucometer (model U-RIGHTTD -4251) to exclude diabetic subjects. Only non-diabetic subjects with blood sugar levels less than 126g/dl were included in this study. Anthropometric measures including height, waist circumference and hip circumference were measured without shoes following the protocol. Under aseptic measures, 5cc of blood samples were drawn for measuring the serum AST, ALT and CK18 levels. After centrifuging the blood at 3000 rev/min for 15-20 minutes, serum was stored in ependendroff tubes at -70ºC in pathology laboratory of King Edward Medical University till samples were run on ELISA.

### Statistical analysis

The data was analyzed using SPSS 21.0. Descriptive were presented as mean ± SD. Normality of data was checked by Shapiro’s wilk test with value ≤ 0.05, showing data is not normally distributed. Regression analysis was applied to explore the association of CK18 with standard liver markers AST and ALT. P-value ≤ 0.05 was considered significant.

## RESULTS

This study comprised of 148 subjects with mean age 44.81 ± 6.2. Out of this 105 (70.9%) were female subjects and 43 (29.1%) were male. The anthropometric and biochemical descriptive of the studied population are shown in Table-I. Significant positive association of CK18 with serum ALT was found (P value 0.05*), but serum AST was not significantly associated with CK18 (P-value 0.29) (Table-II).

**Table-I:** Descriptive Statistics of Studied Subjects (n=148).

Anthropometric and Biochemical Statistics	Mean ± SD	Median (IQR)
Age (years)	44.81 **±** 6.20	43(48-40)
Height (m)	1.59 **±** .06	1.6(1.65-1.57)
Waist (cm)	38.67 **±** 3.66	38(40-38)
Hip (cm)	41.38 **±** 3.95	41(42.0-40.0)
(ALT) (35U/L)	20.04 **±** 20.92	15.0(21.7-11.0)
(AST) (30U/L)	20.99 **±** 25.02	15.0(23.7-9.2)
(CK-18) (U/L)	13.95 **±** 19.84	9.3(14.1-5.1)

**ALT:** Alanine Transaminase, **AST:** Aspartate Transaminase,

**CK18:** Cytokeratin-18, **IQR**: Interquartile range,

**SD:** Standard Deviation, **m:** meter, **cm:** centimeter, **U/L:** units per liter.

**Table-II:** Association of CK18 with Liver Biomarkers.

Independent Variables	Beta Coefficient (ß)	P-value	Confidence Interval
AST	0.051	0.29	-0.117 – 0.219
ALT	1.62	0.05*	-0.153 – 0.249

Dependent Variable: CK18.

## DISCUSSION

NAFLD is a global health issue and its accurate diagnosis is made on liver biopsy. As of care and financial perspectives, liver biopsy is not possible to be done in each patient suffering from NAFLD.^7^ Due to significant rise in the prevalence of NAFLD in concurrence with the remarkable research works for making novel therapies for patients, non-invasive, appropriate and dependable serum biomarkers are really required.^4^ It is noticeable that the laboratory tests done in routine for the assessment of subjects assumed to be NAFLD comprises of a long list of liver biomarkers like serum ALT, AST, alkaline phosphatase(ALP), and gamma-glutamyl-transpeptidase. Various studies have documented that the whole histological picture of NAFLD can be appreciated in patients with normal levels of ALT.^6^ Raised levels of serum ALT and AST might be present in NAFLD patients, though levels within normal do not eliminate the existence of NAFLD and NASH.^7^ When raised, these patients mostly have serum AST to ALT ratios of less than 1, distinct to the alcoholic liver disease in which specifically the ratios are greater indicating that there is no alteration in the degree of advanced fibrosis among normal and the group with raised ALT levels. Furthermore, it is also shown that serum ALT levels two times more than the maximum limit of normal have restricted efficacy in expecting NAFLD with a specificity of 61% and sensitivity of 50%.^1^ (This is the reason that various researches are exploring the diagnostic biomarkers that can be helpful in making the diagnosis. The recent American guidelines provided for making the diagnosis and management of the NAFLD states that serum CK18 levels are useful for diagnostic purpose as well as it also subsidizes in the disease progression. But it is suggested that the usage of CK-18 in routine clinical practice is premature.^8^ Current studies have documented CK18 as convenient biomarker for making the diagnosis of the NAFLD. Musso G also reported similar results concerning CK18 and described it as a biomarker of hepatocyte apoptosis that can be used to evaluate the disease progression.^9^

Moreover, some studies have stated that the accuracy of serum ALT is relatively poor just at 40% for making the diagnosis of NAFLD.^6^ Due to the restrictions of serum transaminases as non-invasive biomarkers of NAFLD, irresistible evidences exhibited that definite cytokines which are derived of various biochemical events comprising of resistance to insulin, oxygen stress, apoptosis and inflammation play significant role in NAFLD progression. Similarly, throughout the hepatocyte apoptosis, the cleaved pieces of CK18 are detectable in patient serum suffering from chronic liver disease. This technique was verified as a capable non-invasive tool in NAFLD/NASH diagnosis.^6^ Swiderska M and his colleges found altered levels of CK18 depending upon the severity of disease inspite of having normal serum ALT.^10^ Incontrast to this, our study found positive association of CK18 with serum ALT. Study conducted by Alt Y and his colleagues are in agreement to our results concerning serum ALT as they reported similar association. However, as regards serum AST they reported contradictory findings to our result as they found significant positive association of serum AST with CK18.^11^

NAFLD exerts considerable negative impact on the patient’s health-related quality. By estimating progression of disease earlier precautionary measures could be adopted to prevent further progression of NAFLD. Noninvasive biomarkers should be applied to clinical practice to reduce unnecessary biopsies.^12^ In future, CK18 can be used for monitoring the disease status as well as evaluating the response to the treatment in NAFLD. Similar researches concerning CK18 and other diagnostic biomarkers should be conducted on a broader scale.^13^

## CONCLUSION

CK18 is positively associated with ALT. Future researches on broader scales are required to validate this association.

### Author` Contribution:

**Benash Altaf** helped in Study design, data collection, Interpretation of results, manuscript writing and is accountable for the integrity of the data.

**Anam Rehman** helped in manuscript writing and drafting, reviewed and approved the manuscript.

**Shireen Jawed** helped in statistical analysis, interpretation of results, formulation of tables, writing the manuscript. Reviewed and approved the manuscript

**Abdul Raouf** helped in manuscript writing and critically reviewed it for intellectually content.
